# Recent advances in cancer detection using dynamic, stimuli-responsive supramolecular chemosensors. a focus review

**DOI:** 10.3389/fchem.2024.1478034

**Published:** 2024-10-07

**Authors:** Kotaro Matsumoto, Keiichi Nakagawa, Daisuke Asanuma, Gaku Fukuhara

**Affiliations:** ^1^ Department of Chemistry, Tokyo Institute of Technology, Tokyo, Japan; ^2^ Department of Bioengineering, The University of Tokyo, Tokyo, Japan; ^3^ Department of Pharmacology, Graduate School of Medicine, The University of Tokyo, Tokyo, Japan

**Keywords:** mechanobiology, supramolecular chemistry, hydrostatic pressure, living cell, chemosensor

## Abstract

In current chemistry, supramolecular materials that respond to a wide variety of external stimuli, such as solvents, temperature, light excitation, pH, and mechanical forces (pressure, stress, strain, and tension), have attracted considerable attention; for example, we have developed cyclodextrins, cucurbiturils, pillararenes, calixarenes, crown ether-based chemical sensors, or chemosensors. These supramolecular chemosensors have potential applications in imaging, probing, and cancer detection. Recently, we focused on pressure, particularly solution-state hydrostatic pressure, from the viewpoint of cancer therapy. This Mini Review summarizes (i) why hydrostatic pressure is important, particularly in biology, and (ii) what we can do using hydrostatic pressure stimulation.

## 1 Introduction

How and to what extent do mechanical stimuli affect biological systems such as living cells and tissues? As a result, it is our long-term dream to fully understand the factors controlling stimuli-induced transitions (growth and differentiation), that is, cell fate determination (Artavanis-Tsakonas et al., 1999; Zhao et al., 2008; Hetz, 2012; Alasaadi and Mayor, 2024). Historically, it has been realized that there may be a relationship between these mechanical forces and living systems from a century ago. The origin is likely to be the proposal in the monumental book, “*On Growth and Form*,” edited by D’Arcy W. Thompson in 1917 (Thompson, 1917), leading to the current “*Mechanobiology*” (Wang and Thampatty, 2006; Ando and Yamamoto, 2009; Ladoux and Mège, 2017; Panciera et al., 2017; Cao et al., 2024). Since the 1990s, the rapid development of force measurements, fine processing, and microscopic techniques has enabled us to answer hypothetical questions in mechanobiology. As a breakthrough discovery in 1999, two groups independently reported that acoustic waves, a generic term for ultrasound and shock waves, could mediate cell characteristics such as morphology and transfection ability (Tachibana et al., 1999; Huber et al., 1999). These findings suggest that, among external stimuli, the propagation and subsequent interaction of dynamic pressure in cells may play a critical role in fate determination. In this Mini Review, we particularly focus on such pressure effects as an external stimulus and introduce some stimuli effects thereafter.

In regenerative medicine, Yamanaka discovered induced pluripotent stem cells (Takahashi and Yamanaka, 2006) and was awarded the Nobel Prize in Physiology or Medicine in 2012. Nevertheless, applying these cells at actual medical sites remains difficult because of the complicated operation of cell culture and the necessity of appropriate scaffolds (Rowland et al., 2010; Melkoumian et al., 2010; Rodin et al., 2010; Miyazaki et al., 2012). Particularly, the mystery relating to the latter has been elucidated. In 2006, Discher *et al.* revealed that the modulus of elasticity in scaffolds can control the growth of cells, that is, fate determination (Engler et al., 2006). This indicates the importance of stimulus induction, which affects cellular morphological changes. Furthermore, a “piezo channel,” a pressure-induced switching aperture, was discovered, in which an applicable influx of calcium ions (Ca^2+^) can be precisely regulated as an appropriate physiological condition (Christensen and Corey, 2007; Coste et al., 2010; Ranade et al., 2015; Murthy et al., 2017). This indicates the significance of pressure stimulation in living systems.

In current mechanobiology, it has been turned out the importance of mutual interactions between a wide variety of external and internal stimuli, such as surface tension, tensile stress, compression force, shearing stress, and pressure (dynamic, osmotic, and hydrostatic) (Wang et al., 2023; Felli et al., 2023; Haydak and Azeloglu, 2024). For example, it was recently realized that shearing stress and extension tension based on blood flow and pressure are highly likely to be factors that regulate the functions of endothelial cells, except for chemical stimulation by hormones (Faller, 1999; Chachisvilis et al., 2006; Sinha et al., 2011; Conway et al., 2013). Looking at the historical background, the recent mainstream of this field appears to have originated from dynamic pressure propagation as an external stimulation. However, visualization, imaging, and quantification of such dynamic pressure in cells have been extremely difficult. Some reasons (Ley et al., 2006; Butcher et al., 2009; Wang and Hu, 2012; Greaves and Maley, 2012) for this are, for example, (a) dynamic pressure waves can be classified into an anisotropic force that often localizes their intensities into specific sites of cells and (b) inherently changes the refractive index of a medium that refracts light toward a high-pressure field. These difficulties seem to hamper further development of mechanobiology, leading us to propose a solution. Hence, we focus on “hydrostatic pressure,” which is one of the isotropic forces in the solution state that is very contrasting from dynamic (or anisotropic) pressure yet easy-to-detectable pressure as an external stimulus (Mizuno and Fukuhara, 2022). It should, therefore, be noted that the relationship between cells and supramolecular chemical sensors or chemosensors (Fukuhara, 2020), upon hydrostatic pressurization, becomes an important object in mechanobiology. In this Mini Review, we summarize our recent progress on hydrostatic pressure effects in living cells and supramolecular chemosensors.

## 2 Hydrostatic pressure effects on HeLa cells

**FIGURE 1:**
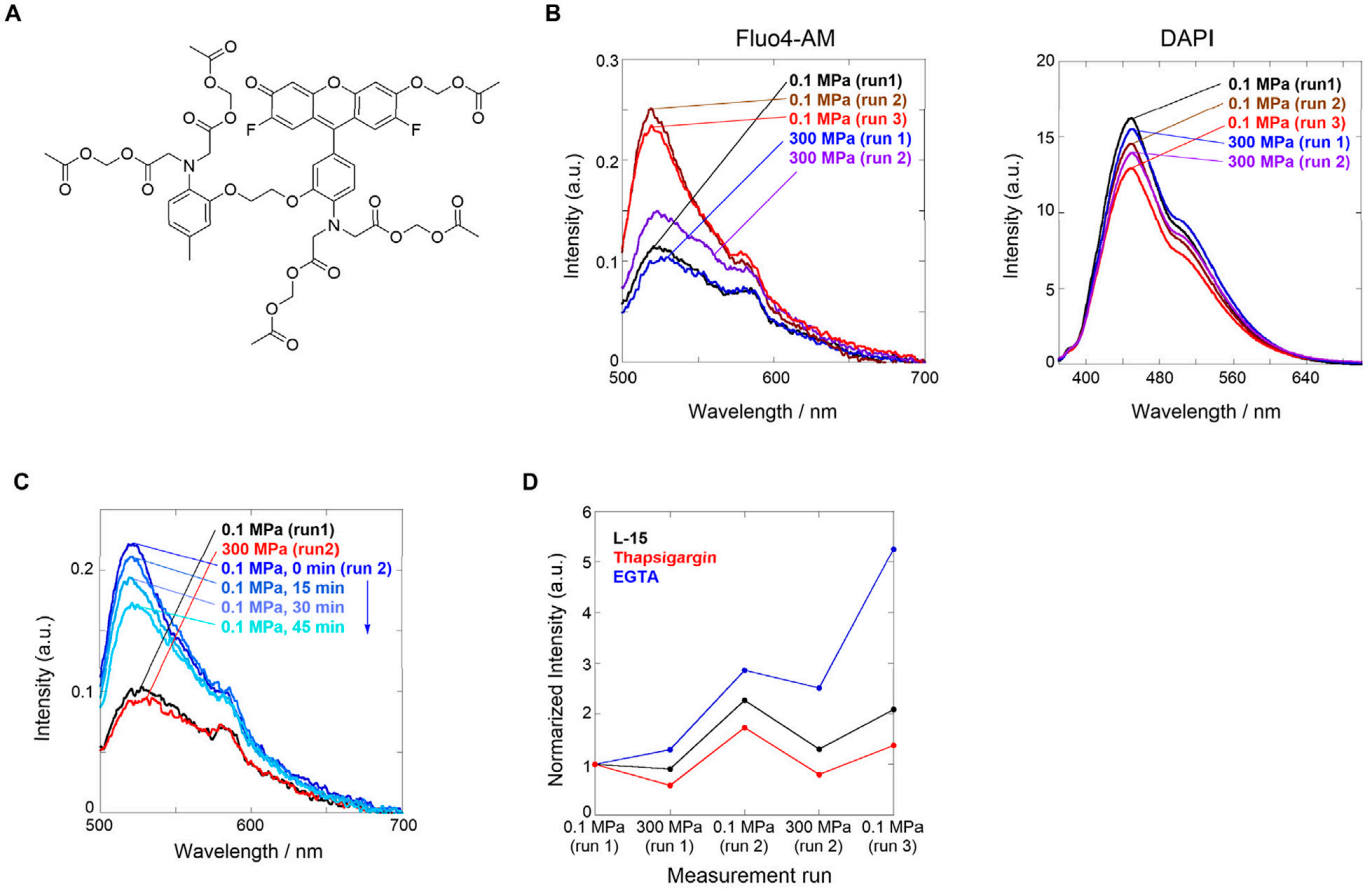
**(A)** Chemical structure of Fluo4-AM. **(B)** Fluorescence spectra of L-15 medium containing HeLa cells loaded with (*left*) Fluor4-AM and (*right*) DAPI. Order of applied hydrostatic pressures: 0.1 MPa (run 1, black) → 300 MPa (run 1, blue) → 0.1 MPa (run 2, brown) → 300 MPa (run 2, purple) → 0.1 MPa (run 3, red). **(C)** Fluorescence spectra of L-15 medium containing HeLa cells stained with Fluor4-AM and DAPI. Order of applied hydrostatic pressures: 0.1 MPa (run 1, black) → 300 MPa (run 2, red) → 0.1 MPa (run 2; 0, 15, 30, and 45 min from blue to sky blue). **(D)** Plots of normalized fluorescence intensities at a peak maximum in the absence (L-15 medium, black) and presence of the inhibitors thapsigargin (red) or EGTA (blue). Reproduced with permission from ref. 36. Copyright 2021 American Chemical Society.

In this section, we present our recent findings regarding hydrostatic pressure regulation applied to tumor cells (Fukuchi et al., 2021). It has been recognized that Ca^2+^ responses through ion channels on the membrane surface are activated by mechanical stimuli such as blood pressure, that is, the abovementioned piezo channel. However, the mechanism that is affected by hydrostatic pressure as a mechanical stimulation is unknown. Thus, elucidating the effect of Ca^2+^ ion channels on hydrostatic pressurization has attracted much attention in mechanobiology. Therefore, we attempted to visualize Ca^2+^ responses in HeLa cells under hydrostatic pressure using Fluo4-AM, which is used as a Ca^2+^-sensitive fluorescent reporter (Figure 1A).

First, we investigated the Ca^2+^ influx response through ion channels in HeLa cells stained with Fluo4-AM upon hydrostatic pressurization. Fluo4-AM is a Ca^2+^-sensitive fluorescent reporter whose fluorescence intensity increases in the presence of Ca^2+^ and, in contrast, decreases under hydrostatic pressure where Ca^2+^ dissociates. Fluo4-AM and DAPI (a dye for elucidating cell death) were loaded into HeLa cells in a cell culture medium (Leibovitz’s L-15), and fluorescence spectra were measured under hydrostatic pressure. DAPI, which can stain dead cells, was selected because of no spectral interference with Fluo4-AM; in the system, only a weak fluorescence was observed at 0.1 MPa. Interestingly, no significant changes in the fluorescence intensity were observed when the hydrostatic pressurization was increased up to 300 MPa, but the fluorescence intensity was enhanced approximately by a factor of two when the hydrostatic pressure was decreased from 300 to 0.1 MPa (Figure 1B, *left*). This finding suggests that hydrostatic pressure stimulation induces the pressure-responsive gate opening of calcium ion channels, causing active Ca^2+^ influx. In contrast, the fluorescence spectrum of DAPI did not change significantly during the pressurization/depressurization cycle (Figure 1B, *right*), suggesting that hydrostatic pressure stimulation did not damage the cell membranes.

During the pressurizing cycle, we observed a time-dependent fluorescence intensity, allowing us to investigate the stability of the pressure-responsive state after depressurization. As described above, HeLa cells stained with Fluo4-AM and DAPI were pressurized to 300 MPa, and the fluorescence spectra were measured every 15 min after depressurization to 0.1 MPa. The fluorescence intensity gradually decreased over time, indicating that Ca^2+^ influx was inhibited step-by-step (Figure 1C). This is highly likely responsible for cell-specific homeostasis, in which the ion channels gated by the hydrostatic pressure stimulation attempt to recover their original state. It should be emphasized that “dynamism” in living cells can be visualized.

Finally, we investigated the origin of the hydrostatic pressurization-regulated Ca^2+^ influx pathways in living cells; which is intracellular or extracellular? The former is a pathway from the endoplasmic reticulum (ER) or a type of organelle, whereas the latter is a pathway through ion channels in the plasma membrane. To confirm this, we performed two contrasting experiments in which the Ca^2+^ influx was inhibited. We used thapsigargin as a blocker of sarco/endoplasmic reticular Ca^2+^-ATPase to deplete it in ER and a chelating ethylene glycol-bis(*β*-aminoethyl ether)-*N*,*N*,*N*′,*N*′-tetraacetic acid (EGTA) as an inhibitor of the influx from the extracellular surface. We applied the hydrostatic pressurization to each HeLa cell. A comparison of both fluorescence intensities upon hydrostatic pressurization showed that the fluorescence intensity was lower after treatment with thapsigargin than after EGTA treatment (Figure 1D). This result led to the conclusion that even after treatment with EGTA, the applicable supply of Ca^2+^ may originate from the intracellular pathway activated by hydrostatic pressure stimulation. Hydrostatic pressurization regulates the dynamism of HeLa tumor cells, causing active Ca^2+^ influx through pressure-responsive ion channels. As described below, these breakthrough findings prompted us to develop stimuli- and pressure-responsive supramolecular chemosensors to visualize living systems under hydrostatic pressurization.

## 3 The first generation of hydrostatic pressure-responsive supramolecular chemosensors: peptide-pyrene conjugates

**FIGURE 2:**
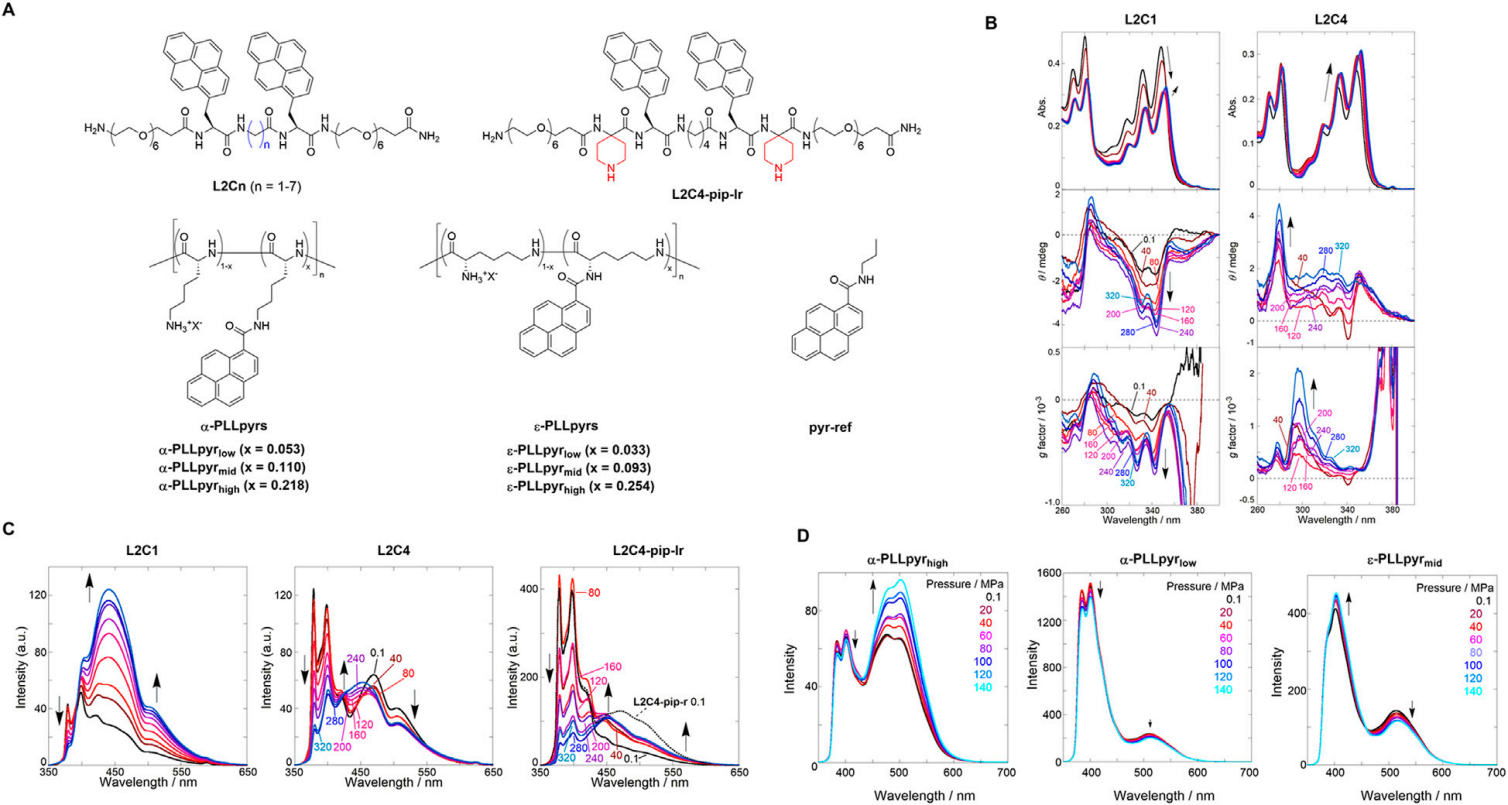
**(A)** Chemical structures of pyrene-based chemosensors. **(B)** UV/vis (top), CD (middle), and *g* factor (bottom) spectra of chloroform solutions of (*left*) **L2C1** and (*right*) **L2C4**. **(C)** Fluorescence spectra of chloroform solutions of (*left*) **L2C1**, (*center*) **L2C4**, and (*right*) **L2C4-pip-lr**. Applied hydrostatic pressure: 0.1, 40, 80, 120, 160, 200, 240, 280, and 320 MPa (from black to sky blue). Reproduced with permission from ref. 37. Copyright 2020 John Wiley and Sons. **(D)** Fluorescence spectra of PBS solutions of (*left*) **α-PLLpyr**_**high**_, (*center*) α**-PLLpyr**_**low**_, and (*right*) **ε-PLLpyr**_**mid**_. Applied hydrostatic pressure: 0.1, 20, 40, 60, 80, 100, 120, and 140 MPa (from black to sky blue). Reproduced with permission from ref. 38. Copyright 2023 American Chemical Society.

Herein, we present the first generation of pressure-responsive oligomeric supramolecular chemosensors comprising two chromophoric pyrenes modified on peptide scaffolds (Mizuno et al., 2020) (Figure 2A, *top*). Pyrene is known to have three major emitting species, depending on its interactions with each other: the first is an emission from a monomer state, the second from an excimer, and the third from a ground-state dimer. These fluorescent species have different emission wavelengths, enabling us to avoid the abovementioned light refraction using the ratiometric fluorescence intensity ratio. According to our guidelines for supramolecular chemosensors, we attempted to quantify the hydrostatic pressure by detecting changes in the fluorescence intensity and chiroptical properties at multiple wavelengths under hydrostatic pressure.

The hydrostatic pressure responses of the two appended pyrenes in **L2C1** and **L2C4** with different methylene numbers were investigated. UV/vis, circular dichroism (CD), and fluorescence spectra were measured for each chloroform solution under hydrostatic pressures ranging from 0.1 to 320 MPa. As shown in Figure 2B (*right*), the absorbance in **L2C4** showed a bathochromic shift and a hyperchromic effect at approximately 350 nm with increasing hydrostatic pressure. These are general pressure-dependent behaviors, the former of which is based on changes in the polarizability of the solvent, and the latter originates only from changes in the effective concentration. The CD and *g* factor (Δε/ε) spectra showed gradual increases at 280–300 nm with elevated pressurization, indicating the induction of a chiral conformational change. As shown in Figure 2B (*left*), the UV/vis spectra of **L2C1**, with the shortest methylene linker, showed a sudden decrease in absorbance with increasing pressure from 0.1 to 80 MPa and then exhibited similar behaviors to those observed in **L2C4** at pressure ranges above 120 MPa. The decrease in absorbance at 0.1–80 MPa was attributed to the strong stacking of pyrenes on the peptide scaffold in the ground state. In the CD spectra, stepwise induction as a negative Cotton effect was observed at 310–350 nm, suggesting that the chiroptical properties of **L2C1** and **L2C4** were quite different upon hydrostatic pressurization.

The fluorescence intensity of **L2C1** decreased in the shorter-wavelength region (350–420 nm), whereas it increased in the medium- (420–500 nm) and longer-wavelength regions (500–650 nm) (Figure 2C, *left*). At an atmospheric pressure of 0.1 MPa, excitation spectra and fluorescence lifetime measurements using the time-correlated single-photon counting method enabled us to characterize the emission species in the three regions as the pyrene monomer (20 ns), ground-state dimer (0.9–1.0 ns), and excimer (24 ns), respectively. In the longer-wavelength region, not only excimer decay but also a rise component (10–12 ns) was observed, which was attributed to “frustrated excimer” or “second excimer,” which is a precursor of excimer. This indicates that the pyrene chromophores tilted slightly to interact with each other in the excited state. With the applied hydrostatic pressure, the *A* factor (relative abundance of the excited species, not population) for the monomer emission decreased from 0.99 to 0.97, while it increased from 0.01 to 0.03 for the ground-state dimer emission. More importantly, the *A* factor ratio (*A*_E_/(−*A*_F_)) between the excimer and frustrated excimer increased from 3.4 to 5.1. It can be concluded that the small distance between the two pyrenes in **L2C1** promotes the formation of ground-state dimers and excimers upon hydrostatic pressurization. As shown in Figure 2C (*center*), the fluorescence intensity of **L2C4** decreased in the shorter-wavelength region (368–390 nm) and the longer-wavelength region (402–650 nm) yet slightly increased in the medium wavelength region (372–475 nm) under hydrostatic pressure. The attributes of the emitted species in the three regions were similar to **L2C1**. These changes, which are very different from **L2C1**, were ascribed to the suppression of the formation of ground-state dimers and excimers separated far from the inter-chromophoric distances. This trend was also observed for other **L2Cn** conjugates (n = 2, 3, and 5–7) under hydrostatic pressure. As shown in Figure 2C (*right*), the hydrostatic pressure response of **L2C4-pip-lr**, in which bulky piperidine substituents were introduced into the peptide scaffold, was also investigated. In contrast to **L2C4**, the fluorescence intensity of **L2C4-pip-lr** increased in the medium- and longer-wavelength regions under hydrostatic pressure and decreased in the shorter-wavelength region. Hydrostatic pressure fluorescence lifetime measurements showed that such an increase was not observed in **L2C4-pip-lr**. This is because the bulky piperidine substituent prevents intermediate stacking of the excited pyrenes.

This study demonstrated that chiroptical and fluorescent properties in response to hydrostatic pressure stimulation can be precisely controlled by changing the distance between the pyrenes on the peptide scaffold or by introducing bulky substituents. We succeeded in visualizing the hydrostatic pressure at three wavelengths as ratiometric responses in the monomer, ground-state dimers, and excimer emissions. This study inspired us to develop supramolecular chemosensors for application in living systems that require water solubility, cellular uptake, and hydrostatic pressure responses.

## 4 The newest type of hydrostatic pressure-responsive supramolecular chemosensor toward application to living systems: polylysine-pyrene conjugates

In the final section, we present our new generation of pressure-responsive supramolecular polymeric chemosensors that employ polylysine, a highly water-soluble and biocompatible cationic polymer, as a scaffold with pyrene as the chromophore (Wakako et al., 2023) (Figure 2A, *bottom*). The hydrostatic pressure response was controlled by varying the degree of substitution (DS) of pyrenes on the polymer backbone.

First, we investigated the hydrostatic pressure response of **α-PLLpyr**_**high**_: α-poly-L-lysine (no methylene linker) as a scaffold was selected with the highest DS as 0.218, and the phosphate-buffered solution (PBS) with an ionic strength of 166 mM was used as a solvent with a view to biological applications. The fluorescence spectra of **α-PLLpyr**_**high**_ in PBS were measured by applying hydrostatic pressure from 0.1 to 140 MPa. As shown in Figure 2D (*left*), a ratiometric response was observed under hydrostatic pressure, with a slight decrease in fluorescence intensity at approximately 400 nm corresponding to monomer emission and an increase in fluorescence intensity at approximately 500 nm corresponding to excimer emission. In **α-PLLpyr**_**low**_ (DS = 0.053) and **α-PLLpyr**_**mid**_ (DS = 0.110), the monomeric and excimer emission intensities decreased with increasing hydrostatic pressure (Figure 2D, *center*). To elucidate the photophysical properties, the hydrostatic pressure fluorescence lifetimes were measured for **α-PLLpyr**_**high**_, which showed six components: 0.2 ns (*τ*_1_), 8.2–9.2 ns (*τ*_2_), and 22–27 ns (*τ*_3_) at 400 nm; 3.1–4.5 (*τ*_4_), 19–25 (*τ*_5_), and 49–54 ns (*τ*_6_) at 500 nm. From comparison with the fluorescence lifetime of the reference compound (**pyr-ref**; see Figure 2A), we can conclude that *τ*_1_ and *τ*_3_ are attributed to the fluorescence of the monomeric state of the pyrene chromophore. The *τ*_1_ species may be explained by a proton transfer process involving the NH groups in the amide moiety. The *τ*_2_ and *τ*_4_ species correspond to the fluorescence of the ground-state dimer. *τ*_5_ and *τ*_6_ species are attributed to the fluorescence of the second excimer (partially overlapped excimer) and the sandwich excimer (fully overlapped excimer), respectively. The ratiometric change in the fluorescence intensity is attributed to an increase in the *A* factor of the sandwich excimer with increasing hydrostatic pressure.

Next, we investigated the hydrostatic pressure responses of **ε-PLLpyr**_**mid**_, a scaffold of ε-poly-L-lysine with a longer methylene linker and medium DS number of 0.093. As shown in Figure 2D (*right*), the fluorescence intensity at approximately 400 nm increased and decreased at approximately 500 nm under hydrostatic pressure. This is the opposite behavior of **α-PLLpyr**_**high**_. The hydrostatic pressure fluorescence lifetime data revealed six components: 0.4–3.1 ns (*τ*_1_), 9.1–11 ns (*τ*_2_), and 20–23 ns (*τ*_3_) at 400 nm; 4.1–5.0 (*τ*_4_), 19–21 (*τ*_5_), and 37–41 ns (*τ*_6_) at 500 nm. The decrease in fluorescence intensity at approximately 500 nm with increasing hydrostatic pressure is attributed to a decrease in the *A* factor by *τ*_6_. The increase in the fluorescence intensity at approximately 400 nm under hydrostatic pressure was due to the suppression of collisional deactivation by solvent attack caused by an increase in the viscosity of the solvent due to pressurization. This study suggests that the fluorescence properties in response to hydrostatic pressure can be controlled by changing the methylene number of the scaffold and DS of the appended pyrenes. More importantly, we developed a new type of supramolecular polymer chemosensor for visualizing hydrostatic pressure stimulation, which operates in a physiological medium such as high-ionic-strength PBS at two ratiometric wavelengths.

## 5 Conclusion

To gain a complete understanding of the long-term mystery of mechanobiology, we focused on *hydrostatic pressure* as one of the wide variety of external and internal mechanical forces that exist in living cells and tissues. In HeLa cells, we observed that the hydrostatic pressure stimulation regulates Ca^2+^ influx through the piezo ion channel, particularly affecting the intracellular pathway. More importantly, we discovered the dynamics of cells attempting to recover their original channel state based on inherent cell homeostasis. These findings hint at an indicator that functions in biological systems, that is, dynamic, stimuli-responsive supramolecular chemosensors, although developing them poses a significant challenge in current chemistry. Different types of peptide scaffolds show distinct hydrostatic pressure responses, particularly various ratiometric fluorescence characteristics, which may lead to new types of supramolecular imaging reagents. These unique features of pressure-responsive supramolecular chemosensors will open new avenues for advances in future mechanobiology. We will cover a further comprehensive summary including other similar systems in a future full review.
